# A Proactive Assessment Framework for Underutilized Species: Evaluating the Bigeye Cigarfish in the Indian Ocean Using a Bootstrap-Coupled Length-Based Bayesian Model

**DOI:** 10.3390/biology15161369

**Published:** 2026-08-11

**Authors:** Guoqing Zhao, Zuozhi Chen, Chao Li, Yongchuang Shi, Fengyuan Shen, Jialiang Yang, Hewei Liu, Ziniu Li, Zhi Zhu, Peng Lian, Lingzhi Li, Hanfeng Zheng

**Affiliations:** 1 Key Laboratory of Oceanic and Polar Fisheries, Ministry of Agriculture and Rural Affairs, East China Sea Fisheries Research Institute, Chinese Academy of Fishery Sciences, Shanghai 200090, China; zgq617717@163.com (G.Z.); bhrock05@sina.com (C.L.); syc13052326091@163.com (Y.S.); fyshen0817@126.com (F.S.); yangjl@eastfishery.ac.cn (J.Y.); hwliu77@126.com (H.L.); lizn@ecsf.ac.cn (Z.L.); dccglc@ecsf.ac.cn (Z.Z.); 2 Key Laboratory for Sustainable Utilization of Open-Sea Fishery, Ministry of Agriculture and Rural Affairs, South China Sea Fisheries Research Institute, Chinese Academy of Fishery Sciences, Guangzhou 510300, China; chenzuozhi@scsfri.ac.cn; 3 Chinese Academy of Fishery Sciences, Beijing 100141, China; lianpeng@cafs.ac.cn; 4 Key Laboratory of Space Ocean Remote Sensing and Applications, Ministry of Natural Resources, Beijing 100081, China

**Keywords:** *Cubiceps pauciradiatus*, Indian Ocean, growth curve, stock assessment, bycatch species

## Abstract

The bigeye cigarfish (*Cubiceps pauciradiatus*) is a common bycatch species in Indian Ocean fisheries, yet its stock status remains largely unevaluated despite its potential as a future “backup” fishery resource. Here, we develop an integrated assessment framework that couples a Bootstrap based growth estimation tool (fishboot) with the Length based Bayesian biomass (LBB) model. Using length frequency data collected from 2023 to 2025, fishboot quantified uncertainty in asymptotic length (*L*_∞_) and growth rate (*K*), yielding estimates of *L*_∞_ = 175 mm and *K* = 0.92 year^−1^ that reflect a fast-growing, medium-bodied life history. This *L*_∞_ was then supplied as an informed prior to the LBB model. Results indicate that the stock maintained a relatively stable resource status throughout the study period, with its baseline biomass remaining healthy (*B*/*B*_msy_ > 1) and no alarming early warning signals detected (*F*/*M* < 1). The modest biomass fluctuation observed is likely attributable to the species’ status as a common bycatch rather than to systematic overfishing pressure. The fishboot–LBB framework provides a robust approach for proactively assessing data limited and bycatch stocks, thereby extending fisheries research beyond traditional target species to support ecosystem-based management in the region.

## Simple Summary

Bigeye cigarfish (*Cubiceps pauciradiatus*) is a common bycatch in Indian Ocean fisheries with potential as a future backup fishery resource, yet its stock status remains largely unassessed. We developed an integrated framework coupling the bootstrap-based fishboot growth estimation tool with the length-based Bayesian biomass (LBB) model. Using 2023–2025 length frequency data, we quantified uncertainty in key growth parameters, confirmed the species’ fast-growing, medium-bodied life history, and applied the estimated asymptotic length as an informed prior to refine the LBB assessment. The stock remained stable throughout the study period, with biomass staying healthily above the sustainable reference threshold and no overfishing warning signals detected; modest biomass fluctuations likely stem from its bycatch nature rather than systematic overfishing pressure. This fishboot–LBB approach provides a robust method for evaluating data-limited and bycatch stocks, expanding fisheries assessment beyond traditional target species to support ecosystem-based fisheries management in the Indian Ocean.

## 1. Introduction

Fisheries represent a critical marine sector in the Indian Ocean and worldwide, serving as an essential source of protein and income for coastal nations [1,2,3]. However, these activities, which typically target highly migratory species, frequently entail the incidental capture of considerable quantities of non-target organisms, commonly referred to as bycatch [4]. To date, the majority of bycatch species lack systematic stock assessment and management, resulting in a limited understanding of their population dynamics and resource status [5,6]. From an ecosystem standpoint, the sustainable use of bycatch species is important not only for their own conservation but also for maintaining the structural stability, functional integrity, and overall resilience of fisheries ecosystems [7,8].

The bigeye cigarfish (*Cubiceps pauciradiatus*) is frequently taken as bycatch in Indian Ocean fisheries. Widely distributed and occurring in considerable biomass, it serves as a mesopredator in regional marine food webs [9,10]. As a mesopredator occupying an intermediate trophic position, the bigeye cigarfish plays a key role in transferring energy from lower to higher trophic levels and contributes to the structural stability of the pelagic food web [9,10]. Currently, this species has limited direct commercial value and is primarily discarded as bycatch. However, given the increasing fishing pressure on principal target species such as tunas and the potential diversification of market demand, it holds promise as a future “backup” fishery resource, especially given rising fishing pressure on primary target species and possible shifts in market demand. For example, several principal target species in the Indian Ocean, such as yellowfin tuna (*Thunnus albacares*) and skipjack tuna (*Katsuwonus pelamis*), have been experiencing elevated fishing pressure in recent years, with some stocks approaching or exceeding sustainable limits. Such conditions create an impetus to explore alternative or complementary fishery resources, including underutilized bycatch species like bigeye cigarfish. Nevertheless, a longstanding lack of species-specific monitoring and study has left fundamental biological parameters and baseline resource data severely lacking. This knowledge gap has effectively placed the species in a management void, impeding the formulation of proactive management strategies [11].

The Length-based Bayesian biomass estimation (LBB) method is widely used to assess data-limited fish stocks [12,13,14]. It derives key management parameters—including optimal length at first capture (*L*_c_opt_) and exploitation rate (*F*)—from length-frequency data, a metric that is often the most feasible to collect for such species [15,16]. Nevertheless, model outputs are sensitive to the choice of prior parameters, notably asymptotic length (*L*_∞_) [12]. In practice, priors are commonly adopted from published studies or assigned based on expert opinion [15]. This introduces substantial prior uncertainty, especially for species with no previous growth information, which can undermine the reliability of stock assessment outcomes. Thus, providing reliable *L*_∞_ priors for species with completely missing data, such as the present study subjects, is a key obstacle and a major source of uncertainty for the application of the LBB model.

Against this background, this study developed an integrated assessment framework based on continuous length monitoring data from 2023 to 2025. The framework combines a bootstrap-based growth parameter estimation method (fishboot) with the LBB model (Supplementary Figure S1). First, fishboot was used to conduct a resampling analysis of the three-year length-frequency time-series data, quantifying uncertainty—particularly in the asymptotic length (*L*_∞_)—and generating probability distributions for the growth parameters. This empirically derived prior information, which explicitly incorporates estimation uncertainty, was then supplied to the LBB model. Together, these steps form a more robust “fishboot-LBB” joint assessment procedure capable of tracking temporal changes in stock status. Using this framework and high-quality time-series data, we systematically analyzed length-structure dynamics of bigeye cigarfish in the Indian Ocean from 2023 to 2025, estimated its population growth parameters, and evaluated three-year trends in resource status. This work provides the first baseline based on a three-year continuous dataset (2023–2025) to support sustainable management of this potential underutilized species.

## 2. Materials and Methods

### 2.1. Study Area and Data Sources

**Figure 1:**
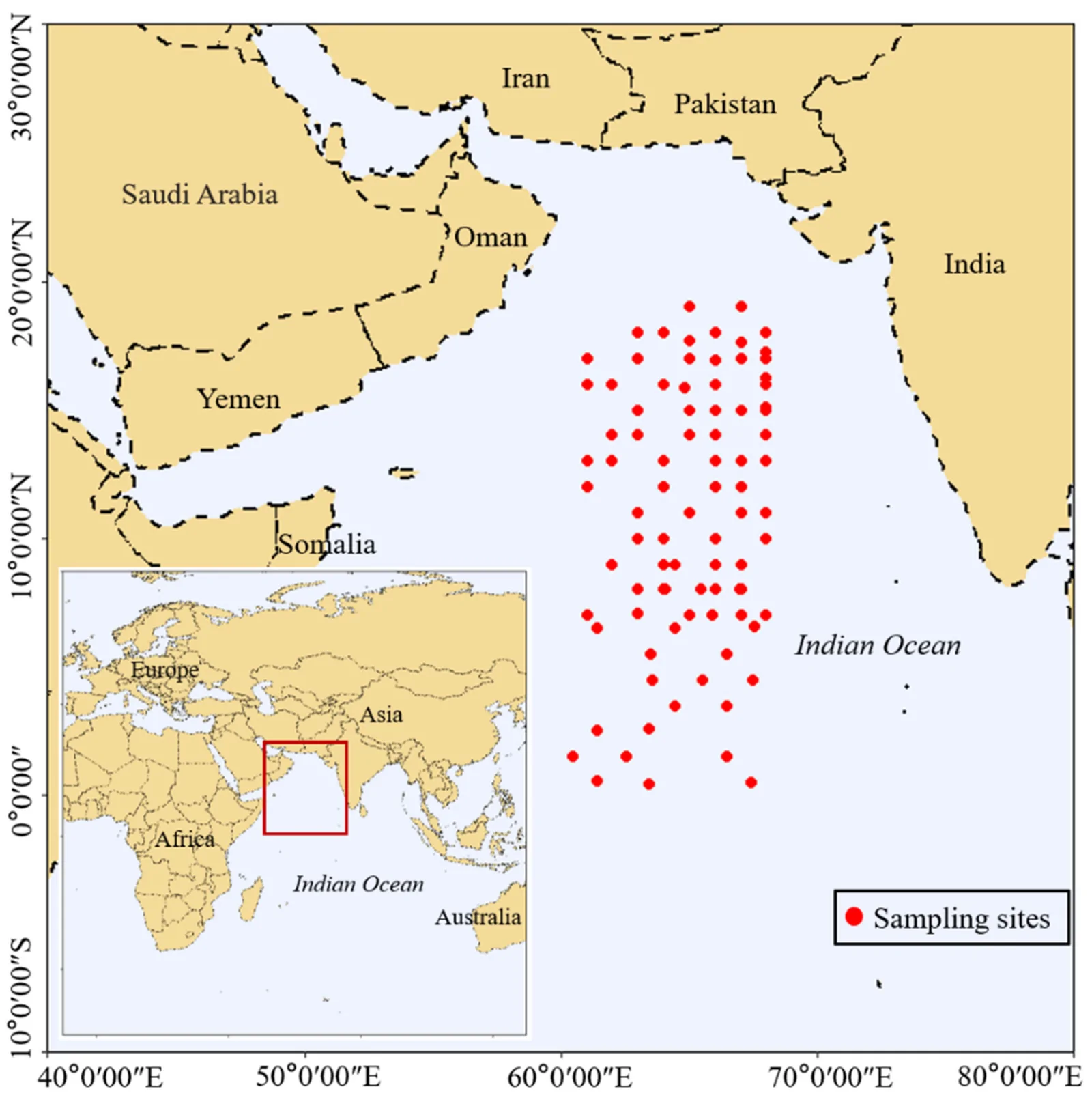
The study area with sampling sites of bigeye cigarfish.

This study focused on the Indian Ocean, where the bigeye cigarfish is widely distributed (Figure 1). The length frequency data used to fit the von Bertalanffy growth equation and the LBB model were obtained from the actual fishery survey (Ministry of Agriculture and Rural Affairs of China: Program on the Survey of Pelagic Fishery Resources), which was sampled from 2023 to 2025. A total of 88 stations were set up (Figure 1, covering a latitudinal range of 0–20° N and a longitudinal range of 60–68° E). Sampling was conducted using a single-boat, four-panel otter trawl with wings and a single codend. The net had a main dimension of 672 m × 163.76 m, with large mesh openings (stretched mesh size 2a = 12 m) in the wing and mouth sections and machine-woven netting in the body section. Two bridles connected the net wings to rectangular otter boards (each approximately 7 m^2^). Towing speed ranged from 3 to 5 knots, and each tow lasted approximately 1 h. The trawl depth was adjusted by varying the warp length based on echo sounder observations to target the fish aggregation layers. The bigeye cigarfish samples were frozen and then taken back to the laboratory for total length measurement. A total of 2645 individuals were collected across 88 sampling stations during the three-year survey period, with annual sample sizes of 771 (2023), 1043 (2024), and 831 (2025). The samples were distributed across stations according to the spatial coverage of the survey cruises, with each station contributing approximately 20–40 individuals on average. During the study period (2023–2025), the tropical Indian Ocean exhibited predominantly positive sea surface temperature anomalies (SSTAs), with the Indian Ocean Dipole mode remaining neutral in 2025 (https://www.cpc.ncep.noaa.gov/products/GODAS/) (accessed on 6 June 2026). These climatic conditions, together with the near-record global ocean warming observed since 2023, may influence the distribution and abundance of pelagic species, including bycatch components such as bigeye cigarfish. However, the present assessment focuses on fishery-dependent length-frequency data, and the potential confounding effects of environmental variability are acknowledged as a limitation.

### 2.2. von Bertalanffy Growth Equation

The von Bertalanffy growth function (VBGF) was fitted to the length-frequency data using ELEFAN-based routines in the TropFishR (1.6.6) [17] and fishboot (1.0.2) [18] R packages, with ELEFAN-based routines using the length frequency data, to estimate the asymptotic length (*L*_∞_) and growth coefficient (*K*) for bigeye cigarfish. The ELEFAN_GA function was applied with the following key settings to optimize the von Bertalanffy growth function (VBGF) parameters: The length-frequency data were aggregated into 10-mm length-class bins. This bin width was chosen as a compromise between retaining sufficient length resolution and ensuring adequate sample sizes per bin for reliable ELEFAN fitting, given the total length range of approximately 43–172 mm observed across all three years; the MA argument was set to 11, corresponding to a moving average over 5 length classes to reduce noise in the data; and seasonal growth oscillation was not considered (M = 0), as its growth in tropical waters is assumed to be relatively unseasonal. The genetic algorithm (GA) was run with a population size of 50 individuals over 100 generations to search for the parameter set (*L*_∞_, *K*, *t*_anchor) that maximized the optimization score (ESP). The genetic algorithm was configured with a population size of 50 individuals and 100 generations. These settings were selected based on preliminary trials showing that a population size of 50 provided sufficient genetic diversity for exploring the parameter space, while 100 generations allowed the algorithm to reach convergence without excessive computational cost. Increasing either parameter yielded negligible improvements in the fit quality (measured by the score function), confirming that the chosen settings were adequate for the present dataset. The best-fitting growth curve identified by ELEFAN_GA was then used as the input for the fishboot function to estimate uncertainty. A non-parametric bootstrap resampling procedure with 1000 iterations was performed, with the random seed set to 123 for reproducibility. The bootstrap samples were generated by resampling the residual distribution of the length-frequency data around the fitted growth curve. The 95% confidence intervals (CI) for each VBGF parameter (*L*_∞_, *K*, *t*_anchor) were calculated as the 2.5th and 97.5th percentiles of the bootstrap distribution. The von Bertalanffy growth equation is given by

(1) $$L_{t}=L_{\infty }(1-e^{-K(t-t_{0})})$$

where *L*_t_ is the length at age *t*, *L*_∞_ is the asymptotic length, *K* is the growth rate (year^−1^), *t*_0_ is the age of the fish at zero length, and *t* is the age of the fish.

### 2.3. LBB Modeling

The model assumes that fish growth is continuous and that maximum body length is attained at the maximum age. This premise allows the body growth rate and natural mortality to be expressed as a constant ratio. Within the LBB framework, the relative rates of natural and fishing mortality (*M*/*K* and *F*/*K*) are used to estimate the ratio of fishing mortality to natural mortality (*F*/*M*) and the ratio of exploited biomass to unexploited biomass (*B*/*B*_0_), thereby reducing the number of required input parameters. Within the LBB framework, the ratio of natural mortality to the growth coefficient (*M*/*K*) is assumed to be constant. This assumption is rooted in the life-history theory of Beverton and Holt, which posits that species with higher growth rates tend to experience higher natural mortality, and that the *M*/*K* ratio is broadly conserved across fish taxa. While this simplification facilitates parameter estimation in data-limited contexts, we acknowledge that *M*/*K* may vary with ontogeny, environmental conditions, and fishing history.

Using length-frequency data as input, the LBB method employs a Bayesian Monte Carlo Markov Chain (MCMC) approach to estimate population parameters. The model estimates the asymptotic length (*L*_∞_), the length at first capture (a gear selectivity parameter, *L*_c_), *M*/*K*, and *F*/*K*. When an independent estimate of *L*_∞_ is available, it is recommended as a fixed input to reduce model uncertainty. In this analysis, the *L*_∞_ value estimated by the fishboot routine was supplied as a fixed input. The detailed derivation is provided by [15], with the main equations summarized below.

Under the input for *L*_∞_, *L*_c_, *M*/*K*, and *F*/*K*, the length at which the unexploited cohort biomass is maximized (*L*_opt_) is calculated using Equation (2) [19]:

(2) $$L_{\mathrm{opt}}=L_{\infty }(\frac{3}{3\;+\;M/K})$$

The optimum length at first capture (*L*_c_opt_) is calculated using Equation (3) [20]:

(3) $$L_{\text{c-opt}}=\frac{L_{\infty }(2\;+\;3F/M)}{(1\;+\;F/M)(3\;+\;M/K)}$$

The yield per recruit (yield-per-recruit, *Y*’/*R*) is given by Equation (4) [21]:

(4) $$Y^{\prime }/R=\frac{F/M}{1\;+\;F/M}{(1-L_{c}/L_{\infty })}^{M/K}(1-\frac{3(1-L_{c}/L_{\infty })}{1\;+\;(\frac{1}{M/K\;+\;F/K})}\;+\;\frac{3{(1-L_{c}/L_{\infty })}^{2}}{1\;+\;(\frac{2}{M/K\;+\;F/K})})\;+\;\frac{{(1-L_{c}/L_{\infty })}^{3}}{1\;+\;(\frac{3}{M/K\;+\;F/K})})$$

The catch per unit effort per recruit (*CPUE*’/*R*) is obtained from Equation (5) [21]:

(5) $${\mathrm{CPUE}}^{\prime }/R=\frac{Y^{\prime }/R}{F/M}=\frac{1}{1\;+\;F/M}{(1-L_{c}/L_{\infty })}^{M/K}(1-\frac{3(1-L_{c}/L_{\infty })}{1\;+\;(\frac{1}{M/K\;+\;F/K})}\;+\;\frac{3{(1-L_{c}/L_{\infty })}^{2}}{1\;+\;(\frac{2}{M/K\;+\;F/K})})\;+\;\frac{{(1-L_{c}/L_{\infty })}^{3}}{1\;+\;(\frac{3}{M/K\;+\;F/K})})$$

The relative biomass per recruit for the unexploited part of the population (*B*_0_′/*R*) is estimated by Equation (6) [21] (*B*_0_′ > *L*_c_):

(6) $${B0^{\prime }/R=(1-L_{c}/L_{\infty })}^{M/K}(1-\frac{3(1-L_{c}/L_{\infty })}{1\;+\;(\frac{1}{M/K})}\;+\;\frac{3{(1-L_{c}/L_{\infty })}^{2}}{1\;+\;(\frac{2}{M/K})})\;+\;\frac{{(1-L_{c}/L_{\infty })}^{3}}{1\;+\;(\frac{3}{M/K})})$$

Subsequently, the relative biomass of the fish population is given by Equation (7) [21]:

(7) $$B/B_{0}={\mathrm{CPUE}}^{\prime }/R{B_{0}}^{\prime }/RB/B_{0}=\frac{{\mathrm{CPUE}}^{\prime }/R}{{B_{0}}^{\prime }/R}$$

When these equations are rerun with *F* = *M* and *L*_c_ = *L*_c_opt_, the relative biomass that produces the maximum sustainable yield (*B*_msy_/*B*_0_) can be estimated. The current biomass relative to the biomass capable of producing maximum sustainable yields (*B*/*B*_msy_) is then calculated using Equation (8) [15]:

(8) $$B/B_{\mathrm{msy}}=\frac{B/B_{0}}{B_{\mathrm{msy}}/B_{0}}$$

## 3. Results

### 3.1. The Growth Parameters of Bigeye Cigarfish

This study analyzed the interannual variations in the length structure of bigeye cigarfish based on length-frequency data collected in the Indian Ocean from 2023 to 2025. The von Bertalanffy growth function was fitted using the bootstrap method to estimate key growth parameters and their uncertainties.

**Figure 2:**
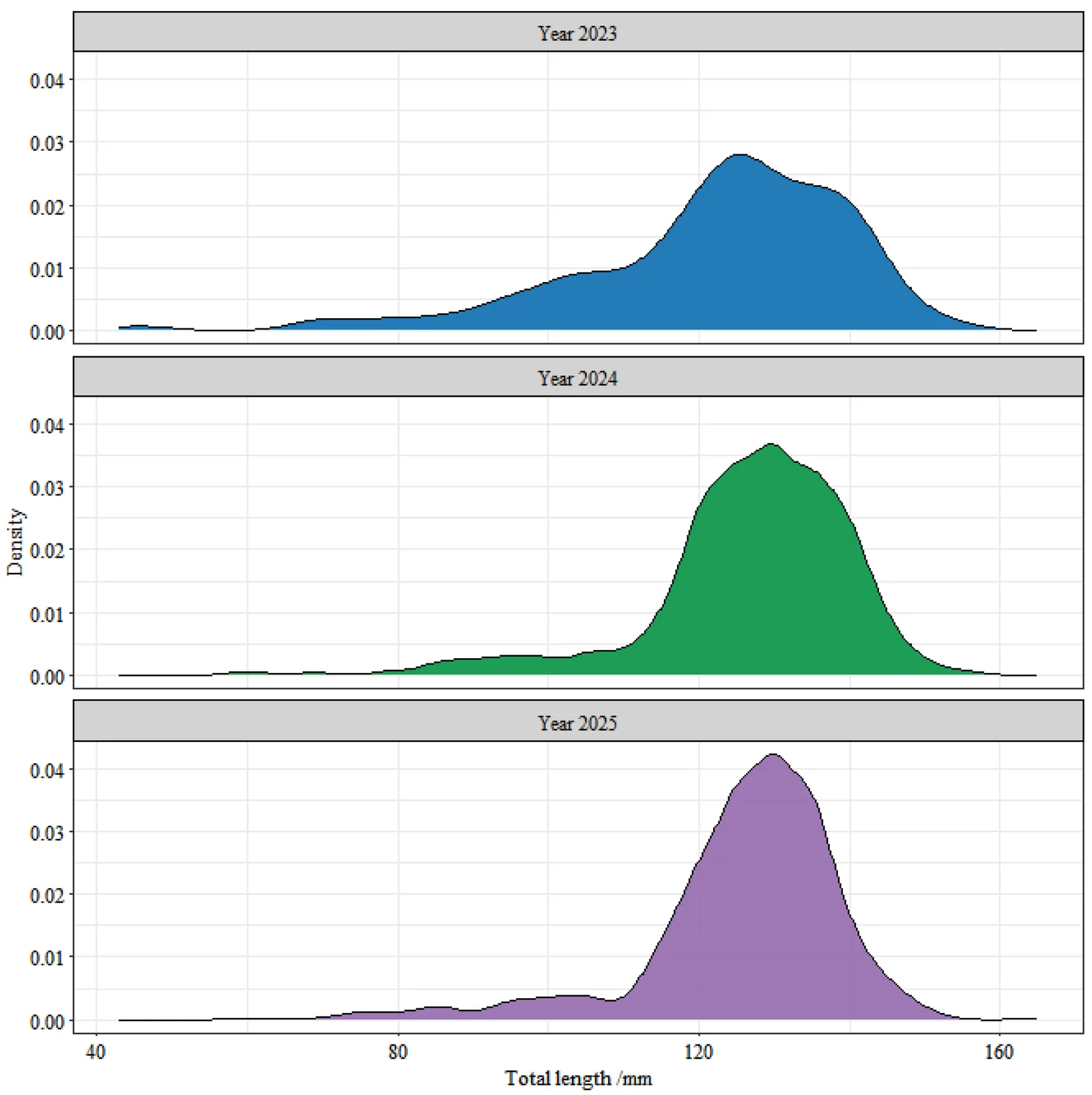
Probability density curve of the length distribution of bigeye cigarfish in the Indian Ocean from 2023 to 2025.

From 2023 to 2025, the length-frequency distribution of bigeye cigarfish exhibited interannual variations (Figure 2). The length distributions in all three years were unimodal, but the peak position and distribution shape differed. In 2023, the length distribution peaked at approximately 125 mm, with a relatively broad curve. In 2024, the main peak shifted slightly to the right, around 130 mm. By 2025, the length distribution changed markedly: the curve narrowed at the base, indicating reduced dispersion of population lengths (Figure 2). The length ranges in different years were comparable, ranging from 43 mm to 172 mm, with the dominant length group between 110 and 140 mm (Figure 2). The annual sample sizes were 771 (2023), 1043 (2024), and 831 (2025) (Table 1), providing a solid basis for interannual comparison.

**Figure 3:**
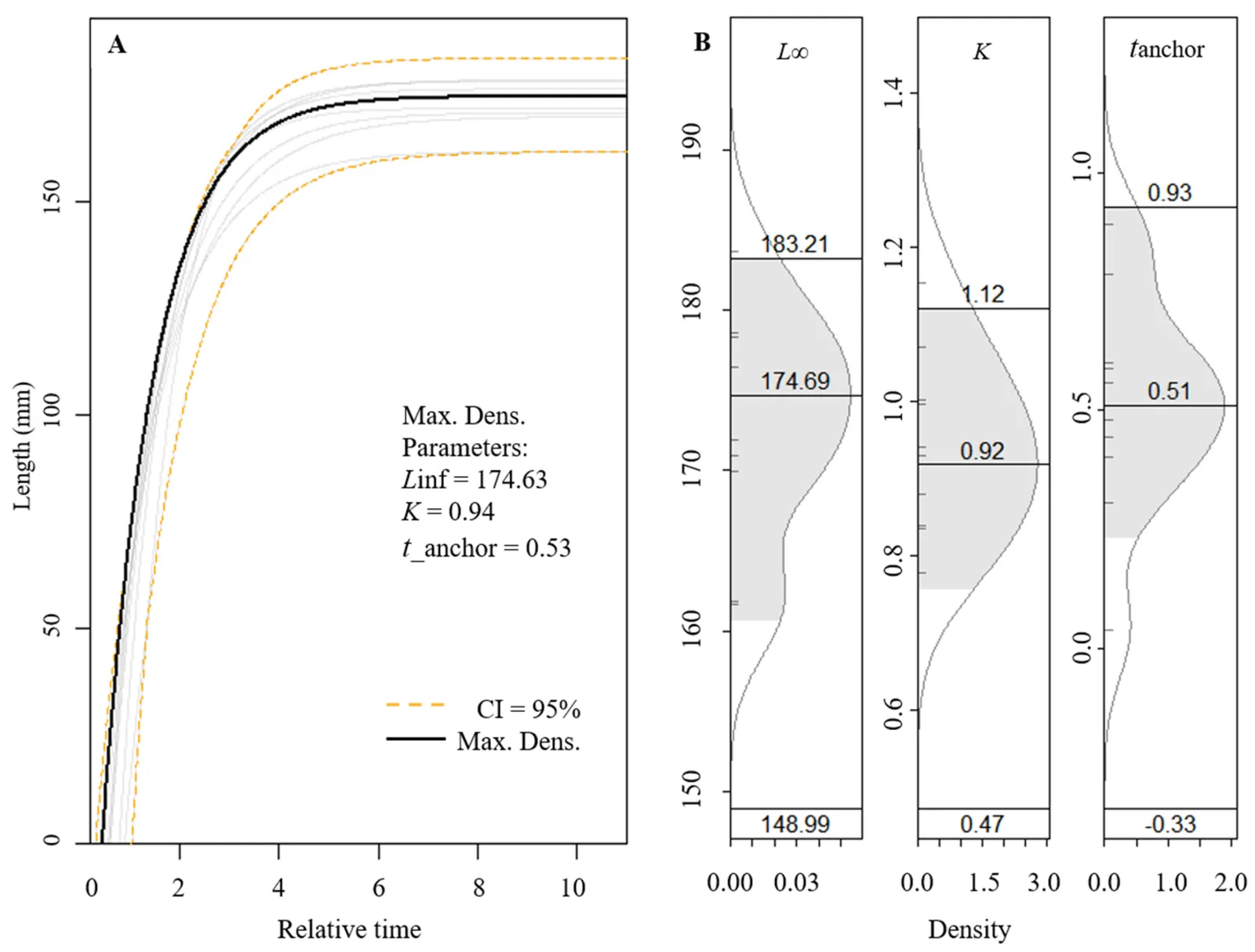
Curve swarms (grey line) and 95% confidence contours (dashed lines) for bigeye cigarfish (**A**) and Kernel density distribution plots and univariate 95% confidence interval of VBGF parameter estimates (**B**).

Growth parameters estimated using TropFishR and fishboot yielded an asymptotic length (L∞) of 175 mm (95% CI: 161–183 mm), a growth coefficient (K) of 0.92 year^−1^ (95% CI: 0.68–1.12 year^−1^), and a theoretical age at zero length (t0) of −0.51 years (95% CI: 0.25–0.93 years) (Figure 3). The resulting von Bertalanffy growth equation was

$$L_{t}=175\times (1-e^{-0.92(t-(-0.51))})$$

These growth parameters, with uncertainties quantified by bootstrap—especially the estimated distribution of L∞—will serve as key prior information for the subsequent LBB assessment model.

### 3.2. LBB Results

Based on the growth parameter priors estimated from TropFishR and fishboot, this study applied LBB to assess the stock status of bigeye cigarfish in the Indian Ocean from 2023 to 2025.

**Figure 4:**
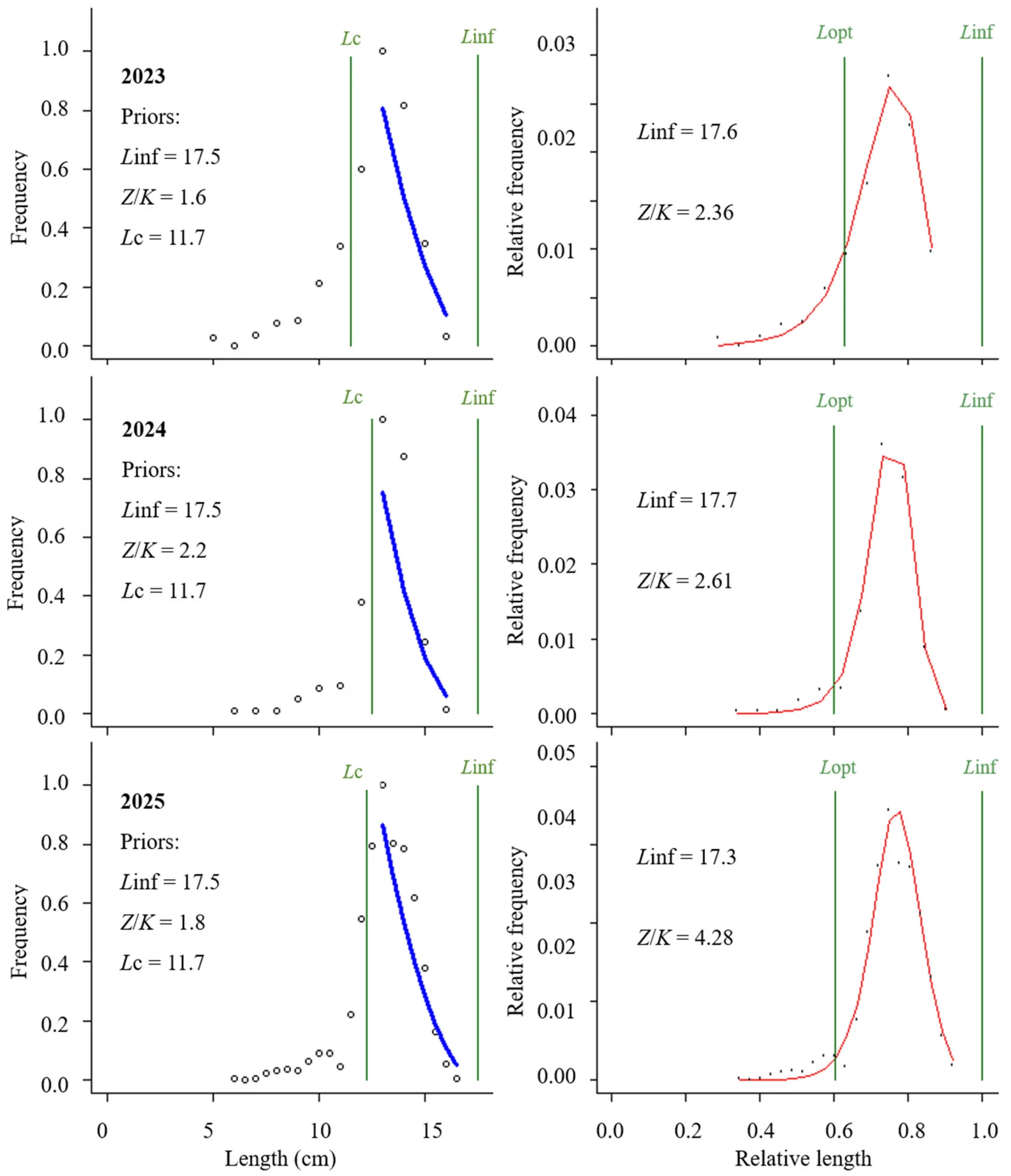
LBB modeling for bigeye cigarfish. *L*_∞_ represents the asymptotic length, *L*_c_ signifies the length at first capture and reflects the selectivity of the fishery, *L*_opt_ represents the length where unexploited cohort biomass was maximized, and *Z*/*K* represents the ratio of total mortality to the somatic growth rate. The relative length frequencies shown in the left panel are used to estimate the prior values of *L_∞_*, *Z*/*K*, and *L*_c_. The blue lines in the left panel depict the prior theoretical growth curves fitted to the input length-frequency data, while the red curves in the right panel represent the posterior fitted trajectories derived from the LBB master equation, which provides the information on *L*_opt_.

The model fitting results for the length data of each year are shown in Figure 4. The left panel presents scatter plots of the length-frequency distributions for the three years, whose trends were used to estimate prior information; the right panel displays the fitting curve of the main LBB equation, which provides the optimal length (*L_opt_*) that maximizes the biomass of an unexploited population. The model runs showed that the prior input value for asymptotic length (*L_∞_* = 175 mm, estimated by fishboot) closely matched the posterior values estimated by the LBB model across all three years (176 mm in 2023, 177 mm in 2024, and 175 mm in 2025; Figure 4, Table 1), with deviations of less than 1.2%. This close agreement verifies the reliability and consistency of the prior information obtained from the fishboot procedure. The fitting curves indicated that although the *L_opt_* values remained relatively stable across the three years, the curve shape changed markedly in 2025, suggesting that the population structure may have undergone significant alteration. Specifically, the length at first capture (*L*_c_) declined from 133 mm in 2023 to 130 mm in 2025 (Table 1), indicating that the fishery is increasingly capturing smaller individuals. Meanwhile, the relative biomass index (*B*/*B*_0_), which reflects resource abundance, showed a decreasing trend from 0.89 to 0.63 over the three years (Table 1). Although this represents a notable decline of approximately 29%, the relative biomass (*B*/*B*_msy_) was estimated to be greater than 1.0 throughout the study period (ranging from 1.8 to 2.6), confirming that the stock remains in a healthy condition (Table 1). Moreover, the B/B_0_ values remained well above the bioeconomic equilibrium level (*B*/*B*_0_ = 0.35 when *F* = *M*), indicating that the observed decline does not constitute a critical depletion of the resource base.

From 2023 to 2025, indicators reflecting fishing pressure showed an increasing trend. The ratio of fishing mortality to natural mortality (*F*/*M*) rose from 0.13 in 2023 to 0.49 in 2025, and the ratio of total mortality to the growth coefficient (*Z*/*K*) increased from 2.36 to 2.92, further confirming the rise in mortality (Table 1). Meanwhile, the relative biomass index (*B*/*B*_0_), which reflects resource abundance, declined continuously from 0.89 to 0.63 (Table 1). Despite the deterioration shown by some indicators, the relative biomass (*B*/*B*_msy_) was estimated to be greater than 1.0, confirming that the stock remains in a healthy condition (Table 1).

## 4. Discussion

### 4.1. Model Uncertainty and Fishboot-LBB Framework Advantages

The LBB model provides clear advantages where data are scarce, yet the robustness of its assessments depends critically on the specification of key prior parameters—above all, the asymptotic length [12]. Conventionally, a single point estimate of *L*_∞_ is often adopted from related species or regional experience [15,16]. Such a “static” prior does not account for the intrinsic uncertainty in parameter estimation. When species with no prior growth information are assessed, this practice can substantially inflate outcome errors and may even lead to an incorrect diagnosis of stock status.

The integrated framework presented here addresses this limitation by incorporating the fishboot approach. Built on bootstrap resampling, fishboot uses actual length-frequency time-series data to estimate the posterior probability distribution of growth parameters, including *L*_∞_. This strategy substantially reduces the assessment risk that stems from an inappropriate or over-confident point-estimate of *L*_∞_. By contrast, had an arbitrarily chosen or expert-opinion-based point prior for *L*_∞_ been used—which could deviate substantially from the true value for a species with no prior growth information—the LBB model outputs for *F*/*M* and *B*/*B*_0_ might have been systematically biased, potentially resulting in an incorrect stock status diagnosis. The fishboot approach mitigates this risk by deriving *L*_∞_ priors empirically from the observed length-frequency data through bootstrap resampling, thereby embedding parameter uncertainty directly into the assessment framework and yielding more defensible stock status classifications. However, we acknowledge that localized, unobserved biases may still exist. For instance, the estimation of key life-history parameters is inherently associated with uncertainty. Furthermore, asymptotic length (*L*_∞_) and the ratio of natural mortality to the growth coefficient (*M*/*K*), as critical model inputs, are estimated with associated confidence intervals.

Nevertheless, the framework offers a replicable and more robust solution for evaluating a wide spectrum of data-limited species. Globally, most bycatch species, emerging fishery resources, and targets of small-scale fisheries face situations analogous to that of bigeye cigarfish: they are characterized by short-term length-frequency data, while critical information such as age structure, fishing-effort time series, or historical abundance indices is absent [22,23]. For such stocks, the technical workflow validated in this study—first quantifying the uncertainty of key biological parameters through resampling, then formally feeding this information as prior input into the assessment model—provides a directly applicable reference.

### 4.2. Data Quality and Model Reliability

The sample size (total *n* = 2645; 771–1043 per year) is sufficient to capture the length structure of bigeye cigarfish within the surveyed region, as the annual length-frequency distributions exhibited clear and consistent modal patterns across all three years. The data were derived from standard commercial fishing gear, ensuring good representativeness of the fishery and effectively capturing the selectivity characteristics of actual fishing operations. During model construction, the catch curves generated by the LBB equations showed a good fit with the observed length-frequency data of bigeye cigarfish across different periods, indicating the reliability and applicability of the estimated model parameters. This consistency underpins the accuracy of the subsequent resource assessment results.

It should be noted, however, that the sampling stations were concentrated in the western tropical Indian Ocean (0–20° N, 60–68° E). Given the wide distribution of bigeye cigarfish across the Indian Ocean basin, this spatial coverage may not fully capture potential regional variations in growth parameters, size structure, or fishing pressure. Consequently, the assessment results presented here should be interpreted as representative of the western Indian Ocean component of the stock rather than the entire basin-wide population. Future surveys extending to other regions (e.g., the eastern Indian Ocean and the Arabian Sea) would help evaluate the spatial consistency of stock status and improve the generalizability of the assessment.

### 4.3. The Population Growth of Bigeye Cigarfish

The estimated growth coefficient for the bigeye cigarfish was high (0.92 year^−1^), characteristic of a fast-growing species. As shown (Table 2), this *K* value is higher than that of most major commercially important fish species in the Indian Ocean, such as bigeye tuna (*K* = 0.31), bluefin tuna (*K* = 0.19), yellowfin tuna (*K* = 0.65), and Indian mackerel (*K* = 0.45), indicating that it has a relatively faster individual growth rate. A high *K* generally enables individuals to attain sexual maturity and a harvestable size early in life, which reduces generation time and can increase population resilience to environmental variability and fishing pressure [24]. The asymptotic length estimated here (175 mm) corresponds to that of a medium-sized pelagic fish, indicating that the species likely occupies an intermediate and relatively stable position in the food web—neither an opportunistic small-bodied taxon nor a large apex predator, but a mid-trophic consumer capable of mediating ecosystem structure and function through both bottom-up and top-down interactions [9,25].

In summary, the bigeye cigarfish displays a “fast-growing, medium-sized” life-history strategy. This trait underpins its potential suitability as a candidate fishery resource: even under increased future exploitation, the population should maintain considerable replenishment and recovery potential, a feature that would support the application of output-based fishery management.

Ecologically, the high growth coefficient positions bigeye cigarfish as an efficient energy conduit within the pelagic food web. Its rapid somatic growth translates into high productivity and turnover rates, enabling it to convert lower-trophic-level production into biomass that is readily accessible to higher predators such as tunas and billfishes. This trophic role, combined with its intermediate body size, suggests that bigeye cigarfish contributes to the stability of the pelagic ecosystem by buffering energy flow fluctuations between trophic levels.

From a fisheries management perspective, the fast-growing life-history strategy provides a biological basis for output-based management approaches, such as total allowable catch (TAC) or quota systems. Species with high *K* values typically exhibit rapid biomass recovery following exploitation, as individuals quickly reach harvestable size and reproduce early. This resilience means that even under moderate fishing pressure, the population can sustain itself through continuous recruitment, making it amenable to catch-based regulations rather than relying solely on input controls (e.g., effort limits). The estimated *K* value of 0.92 yr^−1^, combined with the current healthy stock status (*B*/*B*_msy_ > 1), suggests that output-based management could be a viable option for this species once it transitions from a bycatch to a targeted fishery.

### 4.4. Population Dynamics

The assessment results indicate that the resource status of bigeye cigarfish remained relatively stable during the evaluation period, with only minor fluctuations. The relative biomass (*B*/*B*_0_) declined moderately from 0.89 in 2023 to 0.63 in 2025 but remained well above the bioeconomic equilibrium level (*B*/*B*_0_ = 0.35, when *F* = *M*), and the stock was consistently classified as healthy (*B*/*B*_msy_ > 1). Fishing pressure indicators showed a slight increase, with the exploitation rate (*F*/*M*) rising from 0.13 to 0.49 yet remaining below the critical threshold of 1. This indicates that fishing mortality has not exceeded natural mortality, and there is no evidence of imminent overfishing. As a common bycatch species in Indian Ocean tuna fisheries, the fishing mortality of bigeye cigarfish is largely determined by the overall fishing effort directed at principal target species (e.g., skipjack and yellowfin tuna), rather than by a targeted fishery. The concurrent increase in total mortality (*Z*/*K* from 2.36 to 2.92) and fishing mortality (*F*/*K* from 0.26 to 0.96) likely reflects the intensification of tuna fishing operations in the region during the study period [30], rather than a deliberate increase in exploitation of this species. Importantly, despite this upward trend, the *F*/*M* ratio remained below the critical threshold of 1 throughout the study period, and the stock retained a healthy status (*B*/*B*_msy_ > 1), suggesting that the current level of bycatch pressure has not yet triggered irreversible resource decline.

Notably, no alarming early warning signals—such as a sharp decline in mean length or a collapse in recruitment—were detected. The ratios of mean length to optimal length (*L*_mean_/*L*_opt_) and current length at first capture to optimal length at first capture (*L*_c_/*L*_c_opt_) remained above 1 throughout the study period (1.3–1.4 and 1.4–1.6, respectively), suggesting that the fishery primarily captures individuals that have reached or exceeded the optimal size, with limited direct impact on juvenile cohorts. When the length at first capture exceeds the optimal length, a larger proportion of individuals are allowed to spawn at least once before entering the fishery, thereby safeguarding the reproductive capacity of the stock. This protective effect on the spawning stock helps maintain a steady supply of recruits, even under moderate fishing pressure. In the case of bigeye cigarfish, the current selectivity pattern—targeting mainly medium-to-large individuals—likely buffers the population against recruitment overfishing and contributes to the maintenance of its healthy stock status (*B*/*B*_msy_ > 1). However, the declining trend in *L*_c_ warrants attention, as a continued decrease could eventually push *L*_c_ below *L*_c_opt_, undermining this protective effect.

The modest decline in biomass is likely attributable, at least in part, to the species’ status as a common bycatch in Indian Ocean tuna fisheries [30]. As a non-target species, its incidental capture is influenced by the overall fishing effort directed at principal target species (e.g., tunas), rather than by a targeted fishery. The concurrent increase in *F*/*M* and *Z*/*K* during the study period (Table 1) provides indirect support for the role of fishing pressure in driving the observed biomass decline. However, we acknowledge that environmental factors—such as sea surface temperature anomalies and changes in prey availability—may also contribute to interannual variability in biomass and distribution. Without dedicated environmental covariate analysis, it is not possible to definitively partition the relative contributions of fishing and environment. Future studies integrating oceanographic data (e.g., SST, chlorophyll-a, ENSO indices) with fishery-dependent length-frequency data would help disentangle these effects.

### 4.5. Stock Status and Managements

Despite the declining trend in its resources, the integrated assessment results from the LBB model indicate that the stock is currently (2025) still categorized as “healthy”. The classification of bigeye cigarfish as “healthy” is based on a multi-indicator reference point framework. Specifically, the stock is considered healthy when (i) the relative biomass relative to maximum sustainable yield exceeds unity (*B*/*B*_msy_ ≥ 1), indicating that the current biomass is sufficient to support MSY; (ii) the relative biomass relative to unexploited biomass (*B*/*B*_0_) remains above the bioeconomic equilibrium level (*B*/*B*_0_ = 0.35 when *F* = *M*); and (iii) the exploitation rate remains below the overfishing threshold (*F*/*M* < 1). Throughout the study period, all three criteria were satisfied (*B*/*B*_msy_ = 1.8–2.6, *B*/*B*_0_ = 0.63–0.89, *F*/*M* = 0.13–0.49), collectively supporting the healthy classification. The relatively high growth coefficient (*K* = 0.92 year^−1^) of bigeye cigarfish provides the population with strong potential replenishment and recovery capabilities, thereby allowing it to partially buffer the current fishing pressure (Figure 3, Table 2). Regarding environmental influences, we acknowledge that growth parameters (particularly *K*) and population dynamics can be modulated by environmental conditions such as sea surface temperature, food availability, and oceanographic regime shifts. For tropical pelagic species, warmer temperatures generally accelerate metabolic rates and growth, while food limitation may constrain growth. The present study did not incorporate environmental covariates into the fishboot or LBB models, and therefore the potential confounding effects of environmental variability on the estimated growth parameters and stock status indicators remain unquantified. Future studies that integrate environmental time series (e.g., SST, chlorophyll-a, ENSO/IOD indices) with length-frequency data would help elucidate the relative roles of fishing and environment in shaping the population dynamics of this species. Furthermore, the assessment results also reveal the selectivity characteristics of the primary fishing gears currently in use: the ratios of mean length to optimal length (*L*_mean_/*L*_opt_) and current length at first capture to optimal length at first capture (*L*_c_/*L*_c_opt_) were both greater than 1 over the three years (1.3–1.4 and 1.4–1.5, respectively), indicating that the catch is still dominated by larger individuals that have reached or exceeded the optimal length. This results in relatively limited direct harm to the replenishment group (juveniles), which may be a key reason why a catastrophic decline in the resource has not yet occurred [12]. Therefore, the currently “healthy” resource baseline, the strong potential for growth recovery, and the selective fishing pattern primarily targeting medium-to-large individuals collectively form the biological and fishery foundation for the proactive management of bigeye cigarfish as a “backup development species”.

Based on the assessment findings, targeted management measures are necessary to ensure the sustainability of this resource and realize its exploitation potential. First, implement length-based precautionary management. Model estimates indicate that the current length at first capture (*L*_c_) exceeds the optimal length at first capture (*L*_c_opt_), which is beneficial for resource conservation. It is recommended to clearly set the minimum legal size close to or slightly above *L*_c_opt_ (approximately 90 mm) to ensure adequate growth and reproductive opportunities for the replenishment stock. This measure represents the most direct and effective technical approach to prevent growth of overfishing and maintain the population’s replenishment capacity [31]. Second, control bycatch mortality and exploitation intensity. It is suggested that relevant regional fisheries management organizations list bigeye cigarfish as a specific bycatch species, enabling effective regulation of exploitation intensity through scientific evaluation and limits on its bycatch volume or fishing effort. Third, establish a regular scientific monitoring and assessment system. It is advisable to incorporate this species into regional routine fishery statistics and monitoring programs, systematically collecting its length-frequency and catch data. The integrated “fishboot-LBB” assessment framework validated in this study is suitable for conducting annual or periodic stock assessments, enabling dynamic tracking of stock status changes and providing real-time scientific support for management decisions. Fourth, prospectively explore specialized management models. Should future market demand drive its transition from a bycatch species to a target species, dedicated management regimes—such as output controls based on quotas or input controls based on effort—should be researched and designed in advance. This would establish rules at the early stage of exploitation, ensuring a smooth transition from bycatch to directed fishing and avoiding the passive situation of “exploit first, manage later”.

## 5. Conclusions

This study presents an enhanced assessment framework that integrates the fishboot and LBB models. Using bootstrap resampling to quantify uncertainty in growth parameter estimates, we incorporated this information as prior input into the LBB model, thereby improving the robustness of resource assessments for data-limited species. Results from 2023–2025 length-frequency data show that the Indian Ocean stock of bigeye cigarfish maintained a relatively stable resource status during the evaluation period, with its baseline biomass remaining healthy (*B*/*B*_msy_ > 1) and no alarming early warning signals detected. We provide the first systematic estimates of key growth parameters (*L*_∞_ = 175 mm, *K* = 0.92 year^−1^), revealing a life-history strategy characterized by rapid growth and medium body size, along with the selectivity patterns of current fisheries. The observed modest decline in biomass is likely attributable to the species’ status as a common bycatch in Indian Ocean tuna fisheries rather than to systematic overexploitation. These results suggest the species possesses strong recovery potential and a biological foundation for sustainable exploitation, supporting its proactive management as a candidate “backup” fishery resource. We recommend implementing length-based harvest controls, managing bycatch impacts, and establishing routine monitoring to ensure the long-term sustainability of this stock.

## Figures and Tables

**Table 1:** Descriptive variables concerning the exploitation of bigeye cigarfish in the Indian Ocean from 2023 to 2025.

Index	2023	2024	2025
Sample size	771	1043	831
*L*_∞_ (mm) (user)	175	175	175
*L*_∞_ (LBB/mm)	176	177	175
*L*_c_ (mm)	133	132	130
*M*/*K*	2.04	1.98	1.99
*F*/*K*	0.26	0.64	0.96
*Z*/*K*	2.36	2.61	2.92
*F*/*M*	0.13	0.33	0.49
*L*_mean_/*L*_opt_	1.4	1.3	1.3
*L*_c_/*L*_c_opt_	1.5	1.4	1.6
*B*/*B*_0_	0.89	0.72	0.63
*B*/*B*_0_ (when *F* = *M*)	0.35	0.35	0.35
*B*/*B*_msy_	2.6	2.1	1.8
*Y*’/*R*	0.003	0.010	0.012
Status	healthy	healthy	healthy

*L*_∞_, asymptotic length (the result of fishboot estimation in the previous section was used as input to the LBB model); *L*_c_, length at first capture; *M*, natural mortality; *F*, fishing mortality; *Z*, total mortality; *L*_mean_, the mean length of exploited stock; *L*_opt_, the length where unexploited cohort biomass is maximum; *L*_c_opt_, the optimum length in first capture; *B*, current exploited biomass; *B*_0_, unfished biomass; *B*_msy_, the biomass capable of producing maximum sustainable yields; *Y*’/*R*, yield-per-recruit. Population status classification criteria: healthy, *B*/*B*_msy_ ≥ 1; slightly over-fished, 0.8 < *B*/*B*_msy_ < 1.0; over-fished, 0.5 < *B*/*B*_msy_ < 0.8; grossly over-fished, 0.2 < *B*/*B*_msy_ < 0.5; collapsed, *B*/*B*_msy_ < 0.2.

**Table 2:** Comparison of growth parameter (K) of bigeye cigarfish and important economic fish species in the Indian Ocean in different studies.

Species	Growth Parameter (*K*)	References
bigeye cigarfish	0.92	in this study
Indian oil sardine (*Sardinella longiceps*)	1.57	[26]
skipjack tuna (*Katsuwonus pelamis*)	0.64	[27]
yellowfin tuna (*Thunnus albacares*)	0.65	[27]
bigeye tuna (*Thunnus obesus*)	0.31	[28]
bluefin tuna (*Thunnus thynnus*)	0.19	[27]
Indian mackerel (*Rastrelliger kanagurta*)	0.45	[29]

## Data Availability

The original contributions presented in the study are included in the article, and further inquiries can be directed to the corresponding authors.

## Supplementary Materials

The following supporting information can be downloaded at: https://www.mdpi.com/article/10.3390/biology15161369/s1, Figure S1: Conceptual flowchart of the integrated fishboot-LBB assessment framework.

## References

[1] Andriamahefazafy M., Bailey M., Sinan H., Kull C.A. (2019). The paradox of sustainable tuna fisheries in the Western Indian Ocean: Between visions of blue economy and realities of accumulation. Sustain. Sci..

[2] Beal M., Dias M.P., Phillips R.A., Oppel S., Hazin C., Pearmain E.J., Adams J., Anderson D.J., Antolos M., Arata J.A. (2021). Global political responsibility for the conservation of albatrosses and large petrels. Sci. Adv..

[3] SriHari M., Krishnan P., Sharma R., Mukherjee R., Vivekanandan E. (2025). Status of fisheries management in the Indian Ocean: Key strategies for enhancing sustainability in the Bay of Bengal. Mar. Policy.

[4] Gonzalez-Pestana A., Kouri J.C., Velez-Zuazo X. (2014). Shark fisheries in the Southeast Pacific: A 61-year analysis from Peru. F1000Research.

[5] Durant M., Shackell N.L., Keith D., Tittensor D.P., Worm B. (2025). Addressing bycatch of depleted species through a marine conservation network in the Canadian Atlantic. Can. J. Fish. Aquat. Sci..

[6] Dolman S.J., Brakes P. (2018). Sustainable Fisheries Management and the Welfare of Bycaught and Entangled Cetaceans. Front. Vet. Sci..

[7] Huang C., Rice J., Richter A., Zhou K., Wang Y., Wei C., Pagani-Núñez E., Maleko P.N., Zhang X., Lee T.M. (2024). Effects of fishery bycatch-mitigation measures on vulnerable marine fauna and target catch. Nat. Sustain..

[8] Ayers A.L., Leong K. (2022). Focusing on the human dimensions to reduce protected species bycatch. Fish. Res..

[9] Potier M., Romanov E., Cherel Y., Sabatié R., Zamorov V., Ménard F. (2008). Spatial distribution of *Cubiceps pauciradiatus* (Perciformes: Nomeidae) in the tropical Indian Ocean and its importance in the diet of large pelagic fishes. Aquat. Living Resour..

[10] Lamkin J. (1997). The Loop Current and the abundance of larval *Cubiceps pauciradiatus* (Pisces: Nomeidae) in the Gulf of Mexico: Evidence for physical and biological interaction. Fish. Bull..

[11] Guillou A., Bodin N., Chassot E., Duparc A., Fily T., Sabarros P., Depetris M., Amandè M., Lucas J., Augustin E. (2022). Tunabio: Biological traits of tropical tuna and bycatch species caught by purse seine fisheries in the Western Indian and Eastern Central Atlantic Oceans. Biodivers. Data J..

[12] Wang L., Li J., Kang B. (2022). Fish growth evaluation and fishery dynamic assessment of *Harpadon nehereus* providing insights for its population expansion in the Northern East China Sea. Hydrobiologia.

[13] Zhao G., Feng C., Liu H., Qu T., Fan R., Coker I.C.R., Seisay L.D., Huang H., Li L. (2025). Population Dynamics of Bigeye Grunt Brachydeuterus auritus (Valenciennes, 1831) in the Coastal Waters of Sierra Leone: A Near-Threatened Species on the IUCN Red List. Biology.

[14] Wang K., Zhang C., Ji Y., Xu B., Xue Y., Ren Y. (2025). Navigating life-history parameter uncertainty in data-limited fisheries: A comparative analysis of length-based stock assessment methods. Rev. Fish Biol. Fish..

[15] Froese R., Winker H., Coro G., Demirel N., Tsikliras A.C., Dimarchopoulou D., Scarcella G., Probst W.N., Dureuil M., Pauly D. (2018). A new approach for estimating stock status from length frequency data. ICES J. Mar. Sci..

[16] Froese R., Winker H., Coro G., Demirel N., Tsikliras A.C., Dimarchopoulou D., Scarcella G., Probst W.N., Dureuil M., Pauly D. (2019). On the pile-up effect and priors for Linf and M/K: Response to a comment by Hordyk et al. on “A new approach for estimating stock status from length frequency data”. ICES J. Mar. Sci..

[17] Mildenberger T.K., Taylor M.H., Wolff M. (2017). TropFishR: An R package for fisheries analysis with length-frequency data. Methods Ecol. Evol..

[18] Schwamborn R., Mildenberger T.K., Taylor M.H. (2019). Assessing sources of uncertainty in length-based estimates of body growth in populations of fishes and macroinvertebrates with bootstrapped ELEFAN. Ecol. Model..

[19] Holt S.J. (1958). The evaluation of fisheries resources by the dynamic analysis stocks, and notes on the time factors involved. Int. Comm. Northwest Atl. Fish. Spec. Publ..

[20] Froese R., Winker H., Gascuel D., Sumaila U.R., Pauly D. (2016). Minimizing the impact of fishing. Fish Fish..

[21] Beverton R.J., Holt S.J. (1966). Manual of Methods for Fish Stock Assessment: Part 2-Tables of Yield Functions (Fisheries Report No. 38).

[22] Gray C.A., Kennelly S.J. (2018). Bycatches of endangered, threatened and protected species in marine fisheries. Rev. Fish Biol. Fish..

[23] Komoroske L.M., Lewison R.L. (2015). Addressing fisheries bycatch in a changing world. Front. Mar. Sci..

[24] Hutchings J.A., Myers R.A., García V.B., Lucifora L.O., Kuparinen A. (2012). Life-history correlates of extinction risk and recovery potential. Ecol. Appl..

[25] Cheng C., Su H., Qin Y., Peng Y., Rao Q., Sun S., Li R., Jian Z., Zhong M., Wang Y. (2026). Contrasting effects of producer and consumer resource use efficiency on trophic asynchrony and stability of food web under multiple stressors. Funct. Ecol..

[26] Abdussamad E.M., Rohit P., Ghosh S., Mini K.G., Shameer P., Dipti N.V., Retheesh T.B., Abbas A.M., Akhil A.R., Shihab I. (2022). Age and Growth Studies in Indian Oil Sardine (*Sardinella longiceps*) using Hard Part Microstructure, a Tool for Biological and Ecological Understanding. Thalass. Int. J. Mar. Sci..

[27] Murua H., Rodriguez-Marin E., Neilson J.D., Farley J.H., Juan-Jordá M.J. (2017). Fast versus slow growing tuna species: Age, growth, and implications for population dynamics and fisheries management. Rev. Fish Biol. Fish..

[28] Fu D., DeBruyn P., Fiorellato F., Nelson L., Pierre L., FernandezDiaz C., Chassot E. (2023). Assessing the impact of growth on estimates of fishing mortality-An illustration with Indian Ocean bigeye tuna. Reg. Stud. Mar. Sci..

[29] Saputra S.W., Taufani W.T. (2021). Population parameters and exploitation rate of indian mackerel (*Rastrelliger kanagurta*) on the java’s north coast. AACL Bioflux.

[30] Cieri M., Honneland G., Medley P.A.H., Singh-Renton S. (2025). An Evaluation of the Sustainability of Global Tuna Stocks Relative to Marine Stewardship Council Critenia (Version 12).

[31] Wang L., Lin L., Li Y., Xing Y., Kang B. (2020). Sustainable Exploitation of Dominant Fishes in the Largest Estuary in Southeastern China. Water.

