# Supplementary material for: A Proactive Assessment Framework for Underutilized Species: Evaluating the Bigeye Cigarfish in the Indian Ocean Using a Bootstrap-Coupled Length-Based Bayesian Model
Source: Biology (Basel). 2026 Aug 11;15(16):1369. doi: 10.3390/biology15161369 (PMC13509445; doi:10.3390/biology15161369)
Supplement: Supplementary file 1 [file biology-15-01369-s001.zip › biology-4455746-supplementary.pdf]

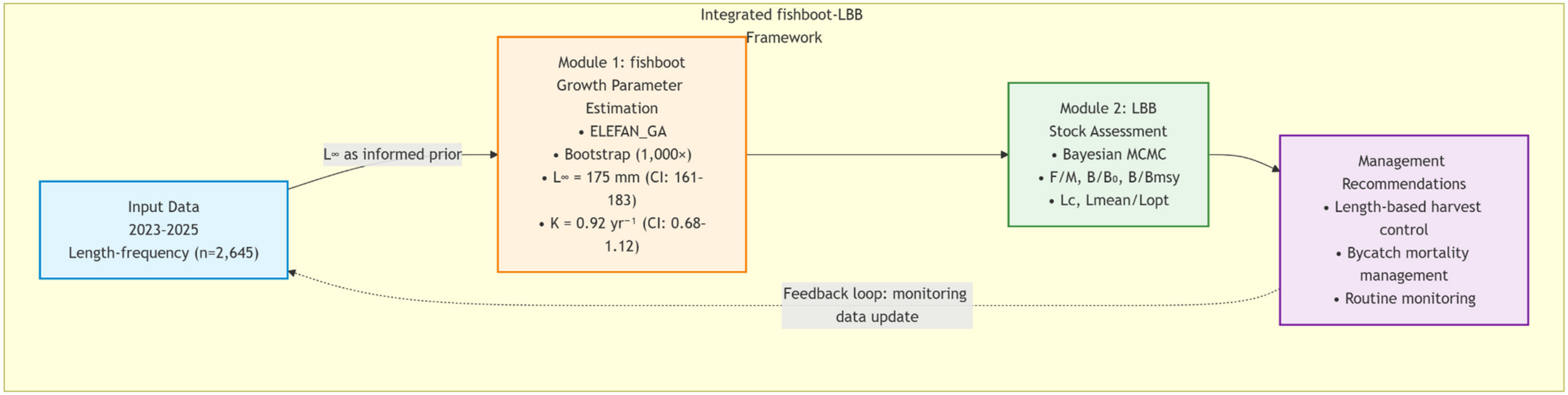

Figure S1. Conceptual flowchart of the integrated fishboot-LBB assessment framework. The framework comprises two analytical modules: (1) the fishboot module, which fits the von Bertalanffy growth function via ELEFAN_GA and quantifies parameter uncertainty through 1,000 bootstrap iterations; and (2) the LBB module, which applies Bayesian MCMC to estimate stock status indicators using the empirically derived $L\infty$ distribution as an informed prior. The dashed arrow indicates a feedback loop whereby ongoing monitoring data can be used to update future assessments.
